# Angiotensin-converting enzyme inhibitors and angiotensin receptor blockers and risk of depression among older people with hypertension

**DOI:** 10.1177/02698811221082470

**Published:** 2022-04-07

**Authors:** Thomas T van Sloten, Patrick C Souverein, Coen DA Stehouwer, Johanna HM Driessen

**Affiliations:** 1 Department of Internal Medicine, Maastricht University Medical Center+, Maastricht, The Netherlands; 2 School for Cardiovascular Diseases (CARIM), Maastricht University, Maastricht, The Netherlands; 3 Division of Pharmacoepidemiology and Clinical Pharmacology, Utrecht Institute of Pharmaceutical Sciences, Utrecht University, Utrecht, The Netherlands; 4 Department of Clinical Pharmacy and Toxicology, Maastricht University Medical Center+, Maastricht, The Netherlands; 5 School of Nutrition and Translational Research in Metabolism (NUTRIM), Maastricht University, Maastricht, The Netherlands

**Keywords:** Depression, hypertension, antihypertensive drugs, pharmacoepidemiology

## Abstract

**Background:**

Angiotensin-converting enzyme inhibitors (ACEIs) and angiotensin receptor blockers (ARBs), commonly used antihypertensive drugs, may have a protective effect against depression in older individuals, but evidence in humans is limited.

**Aims:**

We evaluated the risk of depression, among older individuals with hypertension, comparing ACE or ARB initiators to thiazide(-like) diuretic initiators. Thiazide(-like) diuretics were used as control because these drugs are not associated with mood disorders.

**Methods:**

We used a propensity score-matched new user cohort design with routinely collected data from general practices in England from the Clinical Practice Research Datalink database. We matched 12,938 pairs of new users of ACEIs/ARBs and thiazide(-like) diuretics with hypertension (mean age 67.6 years; 54.7% women). Follow-up time started on the date of drug initiation and ended on the date of treatment discontinuation plus 30 days, or switch to a comparator, occurrence of a study event, death, date of patient’s transfer out of practice, or end of the study period. The primary outcome was a composite endpoint of treated depression and nonfatal and fatal self-harm.

**Results/outcomes:**

Compared to the thiazide(-like) diuretic group, ACEIs/ARBs use was not associated with a lower risk of the primary outcome (hazard ratio 0.96 (95% confidence interval: 0.79; 1.15)). Results did not differ according to lipophilicity, duration of use, and average daily dose, or class (ACEIs or ARBs).

**Conclusions/Interpretation:**

New use of ACEIs or ARBs is not associated with a lower risk of depression among individuals with hypertension.

## Introduction

Depression is a large contributor to global disability in older individuals. Major depression occurs in 2% of adults aged 65 years or older, and its prevalence rises with increasing age (Kok and Reynolds, 2017). The burden related to depression has not satisfactorily decreased in recent decades, despite extensive efforts to treat this disease (Patel et al., 2016). Primary prevention of depression has therefore been identified as a global priority (Cuijpers et al., 2012; Herrman et al., 2019).

Experimental data suggest that angiotensin-converting enzyme inhibitors (ACEIs) and angiotensin receptor blockers (ARBs) may have a protective effect against depression in older individuals via anti-inflammatory and direct neuroprotective effects, and via beneficial effects on the brain vasculature (Culman et al., 2002; Saavedra et al., 2011; Vian et al., 2017). ACEIs and ARBs are widely used for the management of hypertension. However, whether ACEIs, ARBs, or both are protective against the development of depression in humans remains unknown. No randomized clinical trials have investigated the effect of ACEIs or ARBs on the risk of depression. Although some trials have evaluated the effect of ACEIs or ARBs on self-reported depressive symptoms or mental health-related quality of life as a secondary outcome, these studies had inconsistent results with some studies reporting a positive effect (Louis et al., 1999; Palma Gamiz et al., 2006; Pavlatou et al., 2008) and other studies reporting a negative or neutral effect (Brownstein et al., 2018; Leonetti and Salvetti, 1994; Muldoon et al., 2002). In addition, previous observational studies have shown inconsistent results. Some observational studies found an association between the use of ACEIs (Kessing et al., 2020; Williams et al., 2016), ARBs (Cao et al., 2019), or both (Boal et al., 2016) and lower risk of depression in older individuals, whereas other studies (Agustini et al., 2020; Shaw et al., 2021) did not find an association between these drugs and risk of depression. These inconsistent findings could be attributed to differences in study design and choice of outcome measures. In addition, previous studies had some limitations that make it difficult to interpret the results, including incomplete adjustment for important potential confounders, for example socioeconomic status (Boal et al., 2016), lifestyle (Boal et al., 2016; Cao et al., 2019; Kessing et al., 2020; Shaw et al., 2021), or cardiovascular risk factors (Boal et al., 2016; Cao et al., 2019; Kessing et al., 2020; Shaw et al., 2021), prevalent user design (Agustini et al., 2020; Williams et al., 2016) or lack of an active comparator (Boal et al., 2016; Shaw et al., 2021; Williams et al., 2016). Clarifications of the association between ACEIs and ARBs use and risk of depression in observational data could have important implications for mental health: if a protective effect is present in a real-world setting, it would underscore the need for intervention trials.

To address uncertainties in previous research, we aimed to examine a large cohort of individuals with hypertension to evaluate the risk of depression among ACEI or ARB initiators compared to a control group of thiazide(-like) diuretic initiators, using a propensity score-matched cohort design. Initiators of a thiazide(-like) diuretic were used as a control group because these drugs are not associated with mood disorders (Agustini et al., 2020; Boal et al., 2016; Cao et al., 2019; Chevalier et al., 1996; Kessing et al., 2020; Lemogne et al., 2013; Shaw et al., 2021). In addition, we evaluated the risk of depression among ACEI or ARB initiators compared to a control group of dihydropyridine calcium channel blocker (CCB) initiators. CCBs may have beneficial effects on mood (Taragano et al., 2005), and were, therefore, used as a comparator in a secondary analysis.

## Methods

### Data sources

We used the UK Clinical Practice Research Datalink (CPRD) GOLD linked to the Hospital Episodes Statistics (HES) and the Office for National Statistics (ONS) databases.

CPRD-GOLD contains computerized medical records for more than 11 million individuals from 725 practices in the United Kingdom (UK). CPRD currently holds data on ≈7% of the UK population and is generalizable to the UK population (Herrett et al., 2015). CPRD data include patients’ personal characteristics, medical history, laboratory test results, details of prescriptions, specialist referrals, hospital admissions, and major outcomes, with ongoing data collection. Read codes, a hierarchical coding system, are used to define symptoms, diagnoses, referrals, and laboratory or diagnostic tests and results. Read codes are entered by the general practitioner and undergo quality checks before entry into CPRD (Benson, 2011). CPRD data have been validated and shown to be of high quality (Herrett et al., 2011) and used previously to study associations between drugs and depression (Gamble et al., 2018; Hagberg et al., 2016; Jick et al., 2004; Thomas et al., 2013b).

HES data include admission and discharge details of all inpatient and day-case admissions in England and Wales from 1997 onward. HES data include all diagnoses for each episode of care within a hospitalization. The data are validated and cleaned by National Health Service Digital at various stages in the processing cycle before derived fields are added and the data made available for research.

The ONS database contains the electronic death certificates of all UK residents and includes the underlying cause of death (coded using international coding of diseases (ICD)-10).

HES and ONS data are linked to the CPRD since 1998, and linkage is limited to general practices in England that have consented to the linkage scheme (at the time of this study representing 75% of all practices in England).

### Study population

First, we selected all individuals aged 18 years and over in CPRD practices linked with ONS and HES data, with a diagnosis of hypertension in CPRD-GOLD from 1 August 2004 onward. For this study, data collection ended in November 2018. Since August 2004, new National Institute for Health and Care Excellence (NICE) prescribing guidelines for the management of hypertension have been in place (Fiolet et al., 2018). Among this population, we defined two mutually exclusive groups: initiators of an oral ACEI or ARB and initiators of an oral thiazide(-like) diuretic (for a list of included ACEIs, ARBs, and thiazide(-like) diuretics, see Supplemental Material, eTable 1). The date of the first prescription of an ACEI/ARB or thiazide(-like) diuretic defined start of follow-up (index date). Individuals with less than 1 year of continuous enrollment before the start of follow-up were excluded. The 1-year lead-in period minimizes prevalent user bias (Ray, 2003) and improves control for confounding at the index date. The ACEI/ARB and thiazide(-like) diuretic groups were defined as individuals who had not received an ACEI and ARB, or thiazide(-like) diuretic, respectively, within the last 12 months before the index date. Individuals initiating concomitantly an ACEI or ARB and thiazide(-like) diuretic were excluded. According to NICE guidelines from August 2004 onward (Fiolet et al., 2018), thiazide(-like) diuretics are no longer considered a standard first-line treatment for hypertension. In the primary analysis, we therefore required individuals in both groups to have previously used ⩾1 class of an antihypertensive drug other than an ACEI, ARB of thiazide(-like) diuretic before or at the index date. Individuals were allowed to continue to use the other class of an antihypertensive drug after the index date. Next, we excluded individuals with a history of depression, suicidal ideation, suicide attempts or use of drugs for depression, stroke, a malignancy other than nonmelanoma skin cancer, and end-stage renal disease at index date. We also excluded individuals with an indication for an ACEI/ARB other than hypertension, that is, heart failure and diabetic kidney disease before the index date.

### Outcomes

The primary outcome was a composite endpoint of treated depression and nonfatal and fatal self-harm, whichever occurred first, as used previously (Schuerch et al., 2016; Thomas et al., 2013a, 2013b). These were identified from CPRD, using Read and product codes, as well as HES and ONS, using ICD-10 code ranges X60 through X84 and Y10 through Y34. Treated depression was defined as the combination of a Read code for depression and a prescription for a drug for depression within 1 year, as defined previously (Hagberg et al., 2016). The latter of the two dates was used as the event date. The median time between a Read code for depression and a prescription for a drug for depression was 0 days (interquartile range: 0; 34 days). Secondary outcomes were the individual endpoints of treated depression nonfatal and fatal self-harm, any depression (irrespective of new use of drugs for depression), and new use of drugs for depression (irrespective of a new diagnosis of depression).

### Covariates

We selected covariates that are potentially related to depression and hypertension based on previous literature (Aizenstein et al., 2016; Boal et al., 2016; Thomas et al., 2013b; van Sloten et al., 2015a, 2015b). These included age; sex; most recent smoking status (nonsmoker, current smoker, former smoker, undetermined/missing) and body mass index (<20.0, 20.0–24.9, 25.0–29.9, ⩾30 kg/m^2^, undetermined/missing), most recent systolic blood pressure (<120, 120–140, 141–160, >160 mmHg, undetermined/missing) and diastolic blood pressure (<80, 80–90, 91–100, >100 mmHg, undetermined/missing) in the year prior to the index date; and most recent kidney function (based on estimated glomerular filtration rate (eGFR), stages 1–4, undetermined/missing) in the 3 years prior to the index date; any use of lipid-modifying medications and use of psychopharmaceuticals other than drugs for depression (drugs for psychosis, drugs for relapse prevention (oxcarbazepine, carbamazepine, lamotrigine, valproate), drugs for anxiety, and drugs for insomnia) in the year prior to the index date; and lifetime history of psychiatric illness other than depression (mental and behavioral disorders due to psychoactive substance abuse, schizophrenia and related disorders, bipolar disorder, and neurotic, stress-related, and somatoform disorders), or other major chronic illnesses, that is, myocardial infarction, peripheral vascular disease, cerebrovascular disease, cognitive impairment or dementia, chronic pulmonary disease, and diabetes. Kidney function was determined using laboratory test data (reported eGFR) or, if only serum creatinine was available, calculated with the Modification of Diet in Renal Disease (MDRD) formula, or Read codes. In the event of a CPRD Read code and laboratory test result being recorded on the same day, we prioritized the laboratory test result. When multiple values for body mass index were recorded on the same date, we used the highest value. When multiple values for blood pressure or eGFR values were recorded on the same date, we calculated the mean of those values.

### Statistical analysis

We used propensity score matching to control for potential confounding. The propensity score was defined as the predicted probability of patients starting an ACEI/ARB versus a thiazide(-like) diuretic given the aforementioned covariates. We used multivariable logistic regression to estimate the propensity score and matched 1:1 ACEI/ARB initiators to thiazide(-like) diuretic initiators using the nearest neighbor matching algorithm with a matching caliper of 0.02 on the propensity score scale (Austin, 2009). Standardized mean differences were calculated to evaluate the balance after matching.

We summarized data as mean (standard deviation (SD)) for continuous variables and number (percentage) for categorical variables. Incidence rates were calculated for the matched exposure and control groups. Cox proportional hazards models were used to estimate hazard ratios and corresponding 95% confidence intervals (95% CIs) of the outcome with ACEI/ARB initiators compared to thiazide(-like) diuretic initiators. For the primary as-treated analysis, follow-up time started on the index date (date of drug initiation) and ended on the date of treatment discontinuation plus 30 days, or switch to a comparator, occurrence of a study event, death, date of patient’s transfer out of practice, end of data collection by a patient’s practice, or end of the study period, whichever came first. An as-treated analysis was used as the primary analysis because the primary aim of the present study was to investigate the potential protective efficacy of ACEIs and ARBs against the development of depression, that is, among those who completed the treatment. Treatment discontinuation was defined as no new prescription in the 30 days after the expected end date of a prescription, with the expected end date of a prescription calculated based on the dosage instruction and the prescribed quantity. Patients were allowed to enter the study cohort only once. The proportional hazard assumption was assessed by adding an interaction between the exposure and time to the model. Missing data for any covariate were categorized into a separate category and we adjusted for this category in the final model.

### Additional analysis

We evaluated the risk of depression among ACEI/ARB initiators compared to a control group of CCBs initiators, instead of thiazide-like diuretic initiators. For this analysis, we repeated the main analysis among two mutually exclusive groups of individuals aged 18 years or older with hypertension: initiators of an ACEI/ARB and initiators of a CCB. These groups were defined as individuals who had not received an ACEI, ARB, or CCB, within the last 12 months, irrespective of the previous use of other antihypertensive drugs classes.

### Sensitivity analysis

Several sensitivity analyses were performed to test the robustness of the results of the main analysis. First, we did an intention to treat analysis with the initial exposure carried forward to the end of the study period irrespective of treatment discontinuation or switch to a comparator. Second, we repeated the analysis after exclusion of the first 30 days of follow-up to limit the possibility of reverse causality (prescription rates for any drugs, including antihypertensive drugs, might be higher during the onset of depressive symptoms). For this analysis, we repeated the matching procedure after excluding all individuals with an event in the first 30 days of follow-up. Third, we evaluated the risk of depression among ACEI/ARB initiators compared to a control group thiazide(-like) diuretic initiators, irrespective of the previous use of other antihypertensive drugs classes. Fourth, analysis of the primary outcome was performed in six pre-defined subgroups according to age (<55 years, ⩾55 years; because NICE (Fiolet et al., 2018) recommends different antihypertensive drugs according to the age of 55 years), sex, class (ACEIs, ARBs), lipophilicity (lipophilic, less lipophilic; for a list of lipophilic and less lipophilic drugs, see eTable 2), duration of use (1–61, 62–181, 182–364, ⩾365 days), and average daily dose (<0.75, 0.75–1.50, ⩾1.50 of defined daily dose). For these analyses, we divided the follow-up time in intervals of 30 days and updated the stratification variables at the start of each interval. To calculate the average daily dose, the cumulative prescribed dose of all previous prescriptions was reviewed at each interval and defined daily dosages (DDDs) were calculated (www.whocc.no/atc_ddd_index; accessed 12 January 2021) and divided by the time since index date. The study protocol was approved by the Independent Scientific Advisory Committee for Medicines and Healthcare products Regulatory Agency (MHRA) database research, protocol no. 18_136R2.

## Results

**Figure 1.**
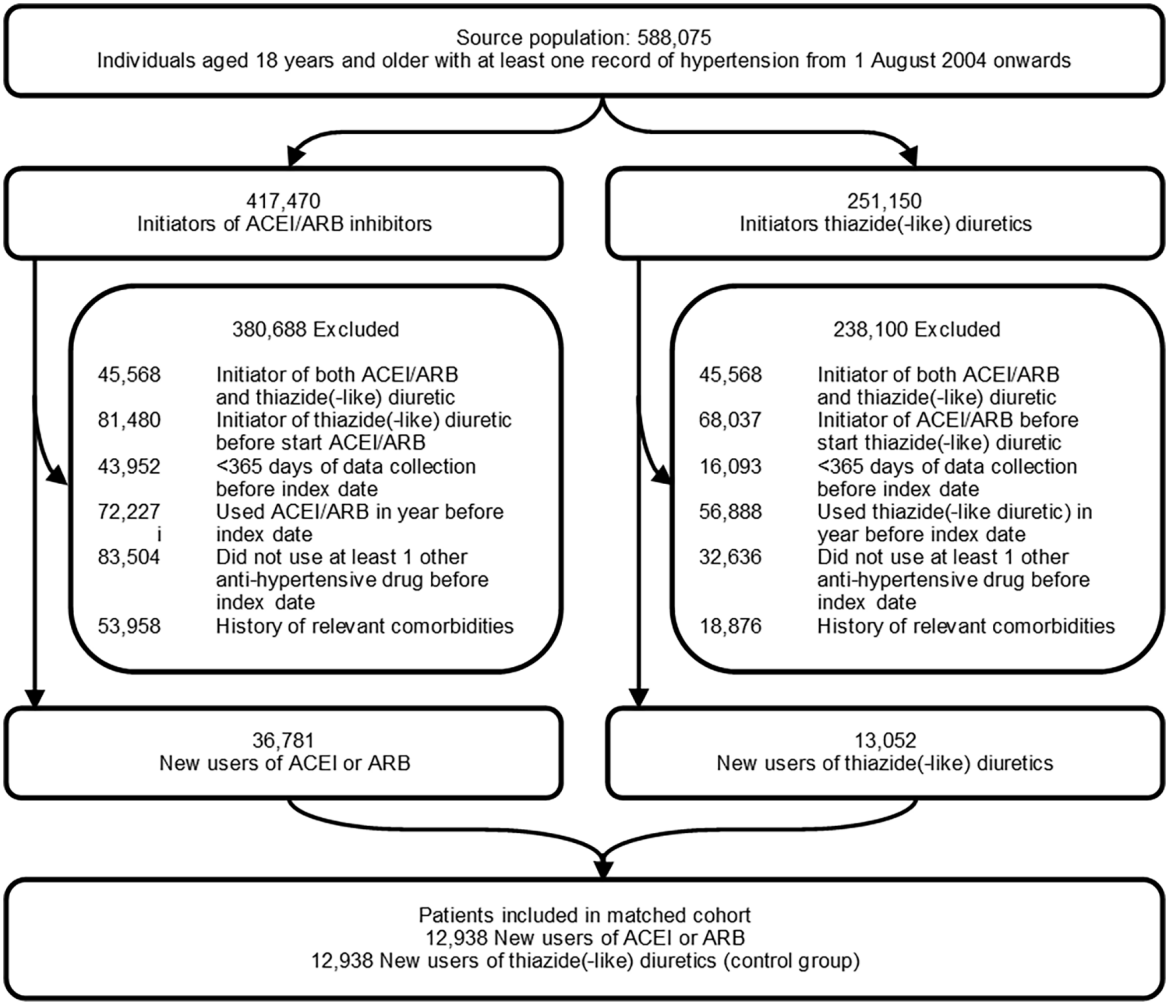
Flow chart of selection process.

Among 36,781 eligible new users of an ACEI/ARB and 13,052 eligible new users of a thiazide(-like) diuretic, 12,938 matched pairs were included in the study cohort (Figure 1). Table 1 describes the baseline characteristics of ACEI/ARB initiators and their matched thiazide(-like) diuretic users (control group) and the standardized mean differences between the two groups. Baseline covariates were well balanced between the two groups after matching on the propensity score. The distribution plots of propensity scores before and after matching are given in eFigure 1. The mean age (SD) of the study population was 67.6 (12.5) years and 54.7% (14,146) were women. Among the ACEI/ARB initiators, a total of 11,505 (88.9%) individuals had initiated an ACEI, 1430 (11.1%) an ARB, and <5 (0.0%) both an ACEI and an ARB at cohort entry. Other antihypertensive drug classes used before or at the index date were a beta-blocker (among 50.2% of ACEI/ARB initiators and 52.6% of thiazide(-like) diuretic initiators), a CCB (among 65.8% of ACEI/ARB initiators and 65.9% of thiazide(-like) diuretic initiators), and any other antihypertensive drug class (among 10.4% of ACEI/ARB initiators and 10.3% of thiazide(-like) diuretic initiators).

**Table 1.** Baseline characteristics in pre-matched and post-matched cohorts.

	Pre-matching			Post-matching		
Characteristics	ACEI/ARB initiators *N* = 36,781	Thiazide(-like) diuretic initiators *N* = 13,052	SMD	ACEI/ARB initiators *N* = 12,938	Thiazide(-like) diuretic initiators *N* = 12,938	SMD
Mean (SD) age (years)	63.0 (13.1)	68.0 (12.6)	−0.35	67.6 (12.4)	67.6 (12.5)	0.00
Women	14,684 (39.9)	7166 (54.9)	−0.30	7088 (54.8)	7058 (54.6)	0.01
ACEI or ARB initiator						
ACEI	32,975 (89.7)	. . .		11,505 (88.9)	. . .	
ARB	3790 (10.3)	. . .		1430 (11.1)	. . .	
Both	16 (0.0)	. . .		<5 (0.0)	. . .	
Smoking status						
None	13,250 (36.0)	5014 (38.4)	0.06	4989 (38.6)	4951 (38.3)	0.03
Current	6100 (16.6)	2190 (16.8)		2042 (15.8)	2174 (16.8)	
Former	16,929 (46.0)	5634 (43.2)		5712 (44.1)	5602 (43.3)	
Missing	502 (1.4)	214 (1.6)		195 (1.5)	211 (1.6)	
Body mass index (kg/m^2^)						
Mean (SD)	28.8 (1.9)	28.0 (5.3)		28.2 (5.4)	28.0 (5.3)	
<20	688 (0.8)	377 (2.9)	0.15	354 (2.7)	363 (2.8)	0.04
20–24.9	7580 (20.6)	3107 (23.8)		2971 (23.0)	3076 (23.8)	
25.0–29.9	13,740 (37.4)	4793 (36.7)		4778 (36.9)	4761 (36.8)	
⩾30	11,841 (32.2)	3510 (26.9)		3675 (28.4)	3500 (27.1)	
Missing	2932 (8.0)	1265 (9.7)		1160 (9.0)	1238 (9.6)	
Systolic blood pressure (mmHg)						
Mean (SD)	153 (21)	157 (20)		157 (20)	157 (20)	
<120	922 (2.5)	177 (1.4)	0.18	172 (1.3)	176 (1.4)	0.02
120–140	9106 (24,8)	2516 (19.3)		2493 (19.3)	2510 (19.4)	
141–160	13,853 (37.7)	5006 (38.4)		5059 (39.1)	4971 (38.4)	
>160	10,314 (28.0)	4432 (34.0)		4355 (33.7)	4369 (33.8)	
Missing	2586 (7.0)	921 (7.1)		859 (6.6)	912 (7.0)	
Diastolic blood pressure (mmHg)						
Mean (SD)	87 (12.8)	87 (12)	0.05	87 (12)	87 (12)	0.03
<80	8126 (22.1)	922 (2.5)		2819 (21.8)	2840 (22.0)	
80–90	14,395 (39.1)	9106 (24.8)		5433 (42.0)	5259 (40.6)	
91–100	7480 (20.3)	13,853 (37.7)		2567 (19.8)	2603 (20.1)	
>100	4203 (11.4)	10,314 (28.0)		1260 (9.7)	1324 (10.2)	
Missing	2577 (7.0)	2586 (7.0)		859 (6.6)	912 (7.0)	
Chronic kidney disease						
Stage 1	5912 (16.1)	1540 (11.8)	0.19	1469 (11.4)	1537 (11.9)	0.03
Stage 2	19,921 (54.2)	7057 (54.1)		7170 (55.4)	7028 (54.3)	
Stage 3	6225 (16.9)	2087 (16.0)		2125 (16.4)	2086 (16.1)	
Stage 4	151 (0.4)	24 (0.2)		23 (0 2)	24 (0 2)	
Missing	4572 (12.4)	2344 (18.0)		2151 (16.6)	2263 (17.5)	
Medication use						
Use of lipid–modifying drugs	11,865 (32.3)	3192 (24.5)	0.17	3138 (24.3)	3187 (24.6)	−0.01
Use of psychopharmaceuticals other than drugs for depression^a^	2360 (6.4)	1000 (7.7)	−0.04	920 (7.1)	980 (7.6)	−0.01
Any psychiatric illness other than depression^b^	150 (0.4)	66 (0.5)	−0.01	63 (0.5)	63 (0.5)	−0.00
Myocardial infarction	2165 (5.9)	224 (1.7)	0.22	216 (1.7)	224 (1.7)	−0.00
Peripheral vascular disease	457 (1.2)	167 (1.3)	−0.00	155 (1.2)	164 (1.3)	−0.01
Cerebrovascular disease	225 (0.6)	77 (0.6)	0.00	66 (0.5)	77 (0.6)	−0.01
Dementia	99 (0.3)	63 (0.5)	−0.03	48 (0.4)	59 (0.5)	−0.00
Chronic pulmonary disease	1157 (3.1)	490 (3.8)	−0.03	454 (3.5)	484 (3.7)	−0.01
Diabetes mellitus	4340 (11.8)	464 (3.6)	0.31	470 (3.6)	464 (3.6)	−0.00

ACEI: angiotensin-converting enzyme inhibitor; ARB: angiotensin receptor blocker; SD: standard deviation; SMD: standardized mean difference.

Values are numbers (percentages) unless stated otherwise.

a Drugs for psychosis, drugs for relapse prevention (oxcarbazepine, carbamazepine, lamotrigine, valproate), drugs for anxiety, and drugs for insomnia.

b Mental and behavioral disorders due to psychoactive substance abuse, schizophrenia and related disorders, bipolar disorder, and neurotic, stress-related, and somatoform disorders.

**Figure 2.**
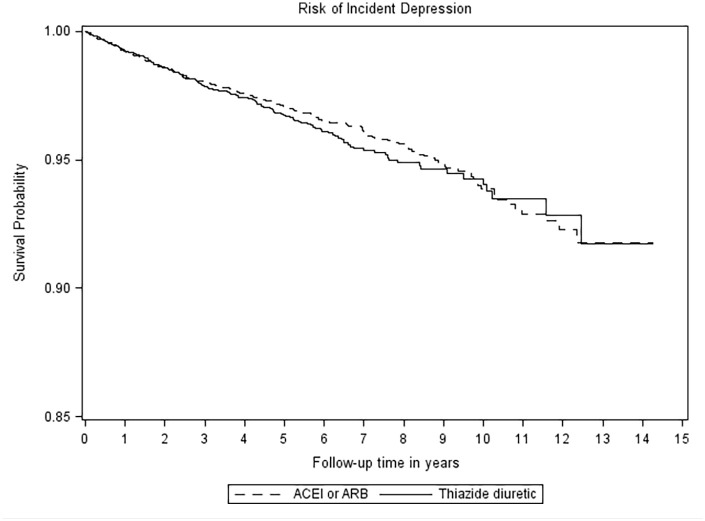
Kaplan–Meier curve of primary outcome-treated depression and nonfatal and fatal self-harm.

The median duration of follow-up in the matched cohort for the composite primary outcome was 2.1 (interquartile range 0.5–5.7) years in the ACEI/ARB group and 0.9 (interquartile range 0.2–3.5) years in the control group, generating a total of 44,295 and 29,939 person years of observation time, respectively. Main reasons for the end of follow-up were treatment discontinuation (for ACEI/ARB initiators 23.4% and thiazide(-like) diuretic initiators 21.5%), switch to comparator (for ACEI/ARB initiators 20.9% and for thiazide(-like) diuretic initiators 46.1%), death (for ACEI/ARB initiators 7.4% and thiazide(-like) diuretic initiators 4.6%) or other reasons (i.e. date of patient’s transfer out of practice, end of data collection by a patient’s practice, or end of the study period; for ACEI/ARB initiators 48.3% and for thiazide(-like) diuretic initiators 27.8%). Figure 2 shows the Kaplan–Meier curves for the primary outcome composite endpoint of treated depression and nonfatal and fatal self-harm. Table 2 shows the incidence rates, absolute rate differences, and hazard ratios for the primary and secondary outcomes. During follow-up, a total of 467 (0.02%) cases of the primary outcome occurred. The incidence rate in the ACEI/ARB group was 6.2 per 1000 person years (272 cases of depression) and in the thiazide(-like) diuretic group 6.6 per 1000 person years (195 cases of depression). Compared to the control group, new use of an ACEI or ARB was not associated with a lower risk of the primary outcome-treated depression and nonfatal and fatal self-harm (absolute rate difference −0.4 (95% CI −1.6; 0.8), hazard ratio 0.96 (95% CI 0.79; 1.15)). In addition, new use of ACEI or ARB was not associated with a lower risk of the secondary outcome-treated depression, any depression, and new use of drugs for depression. The secondary outcome fatal and nonfatal self-harm could not be evaluated due to low number of events (8 in the ACEI/ARB group and <5 in the control group).

**Table 2.** Incidence rates and hazard ratios for incident depression for angiotensin-converting enzyme inhibitors (ACEIs) or angiotensin receptor blockers (ARBs) initiators compared to controls.

Exposures	Incidence rate per 1000 person years (95% CI)	Absolute rate difference per 1000 person years (95% CI)	Hazard ratio (95% CI)
**Composite primary outcome** ^a^			
Controls (thiazide(-like) initiators)	6.6 (5.8; 7.6)	0 [Reference]	1 [Reference]
ACEI/ARB initiators	6.2 (5.6; 7.0)	−0.4 (–1.6; 0.8)	0.96 (0.79; 1.15)
**Secondary outcomes**			
*Treated depression*			
Controls (thiazide(-like) initiators)	6.6 (5.7; 7.6)	0 [Reference]	1 [Reference]
ACEI/ARB initiators	5.9 (5.3; 6.7)	−0.6 (–0.5; 1.8)	0.91 (0.76; 1.10)
*Nonfatal and fatal self-harm*			
Controls (thiazide(-like) initiators)	0.1 (0.0; 0.3)	0 [Reference]	1 [Reference]
ACEI/ARB initiators	0.4 (0.2; 0.6)	0.3 (0.1; 0.6)	n/a
*Any depression (irrespective of new use of drugs for depression)*			
Controls (thiazide(-like) initiators)	8.0 (7.1; 9.1)	0 [Reference]	1 [Reference]
ACEI/ARB initiators	7.2 (6.4; 8.0)	−0.9 (–2.2; 0.4)	0.91 (0.77; 1.08)
*New use of drugs for depression (irrespective of depression diagnosis)*			
Controls (thiazide(-like) initiators)	33.6 (31.5; 35.8)	0 [Reference]	1 [Reference]
ACEI/ARB initiators	31.1 (29.5; 32.8)	−2.5 (–5.2; 0.2)	0.94 (0.86; 1.02)

ACEI: angiotensin-converting enzyme inhibitor; ARB: angiotensin receptor blocker; CI: confidence interval.

a Composite end point of treated depression and nonfatal and fatal self-harm.

### Additional analysis

For this analysis, 41,921 matched pairs of ACEI/ARB initiators and CCB initiators were included. Baseline covariates were well balanced between the two groups after matching on the propensity score (data not shown). The mean age (SD) of the study population was 64.3 years and 54.2% (*n* = 37,911) were women. During follow-up, a total of 1392 (0.02%) cases of the primary outcome occurred. The incidence rate in the ACEI/ARB group was 6.3 per 1000 person years (844 cases of depression) and in the CCB group 5.5 per 1000 person years (548 cases of depression). Compared to the control group, new use of an ACEI or ARB was associated with higher risk of the primary outcome-treated depression and nonfatal and fatal self-harm (absolute rate difference 0.8 (95% CI 0.1; 1.4), hazard ratio 1.16 (95% CI 1.04; 1.30)).

### Sensitivity analysis

**Figure 3.**
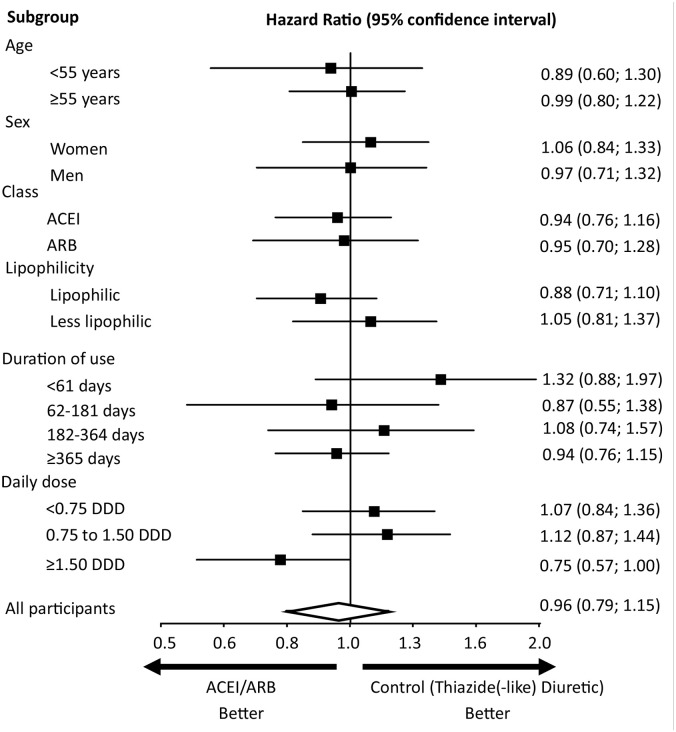
Incidence of primary outcome treated depression and nonfatal and fatal self-harm according to subgroups. ACEI: angiotensin-converting enzyme inhibitor; ARB: angiotensin II receptor antagonist; DDD: defined daily dose. For lipophilic and less lipophilic ACEIs/ARBs, please see Supplemental Material, eTable 2.

The intention-to-treat analysis, the analysis with exclusion of the first 30 days of follow-up, and the comparison of groups of ACEIs/ARBs initiators and thiazide(-like) diuretic initiators irrespective of previous use of antihypertensive drugs provided results that were consistent with those obtained in the main analysis (eTable 3). The results across the six pre-defined subgroups are shown in Figure 3. Results according to the subgroups were similar to those obtained from the total study population.

## Discussion

In this large study of older individuals with hypertension, we did not find a lower risk of depression associated with the initiation of ACEI or ARB compared with initiating use of negative control (a thiazide(-like) diuretic).

Data in humans on the protective effect of ACEIs or ARBs for depression are limited. We are not aware of randomized clinical trials of ACEIs or ARBs and risk of depression. In addition, some previous observational studies have examined the association between ACEIs or ARBs use and risk of depression (Agustini et al., 2020; Boal et al., 2016; Cao et al., 2019; Kessing et al., 2020; Shaw et al., 2021; Williams et al., 2016) and these studies had inconsistent findings and several limitations. A study that used a large hospital database of 525,046 individuals from Scotland found that individuals that used an ACEI or an ARB had a lower risk of mood disorders compared to individuals using no or other antihypertensive drug classes (Boal et al., 2016). In addition, a nested-case control study from Australia among 954 individuals found that ACEIs were used less often in individuals with depression compared to those without depression (Williams et al., 2016). Also, a study that used a large insurance database from Beijing, China, and included 181,709 individuals with a new diagnosis of hypertension found that ARB use was associated with a lower risk of depression compared to the use of other antihypertensive drug classes (Cao et al., 2019). In contrast, another study from Scotland that used linked healthcare data and included 538,730 individuals (Shaw et al., 2021) and a secondary analysis from the ASPirin in Reducing Events in the Elderly (ASPREE) trial (Agustini et al., 2020), which investigated the effects of aspirin on several endpoints among 14,195 individuals from Australia and the United States, did not find an association between ACEI or ARB use and incident depression and presence of depressive symptoms, respectively. Finally, a study that used linked healthcare data from Denmark found that among 3747,190 individuals, a lower risk of depression was observed for only two (enalapril and ramipril) of 16 evaluated ACEIs or ARBs (Kessing et al., 2020). Most of these studies adjusted for only a limited number of potential confounders (Boal et al., 2016; Cao et al., 2019; Kessing et al., 2020; Shaw et al., 2021), combined new and prevalent ACEI or ARB users (i.e. prevalent user design) (Agustini et al., 2020; Boal et al., 2016; Williams et al., 2016), or had a reference group of individuals that did not use any antihypertensive medications (nonusers) (Boal et al., 2016; Shaw et al., 2021; Williams et al., 2016). These limitations increase the risk of bias, notably bias due to confounding by indication (Ray, 2003). The study from Kessing et al. compared the risk of depression among individuals that had used a higher number of ACEI or ARB prescriptions to individuals with a lower number of prescriptions (Kessing et al., 2020). Such comparison could, however, have been affected by immortal time bias, which tends to exaggerate effectiveness (Levesque et al., 2010).

Animal and in vitro studies that suggest that increased renin–angiotensin system activity may affect affective and cognitive processing via multiple mechanisms. Increased renin–angiotensin system activity has been linked to neuro-inflammation, blood–brain barrier leakage, and reduced cerebral microvascular perfusion in regions responsible for emotional behaviors (Ando et al., 2004; Trauernicht et al., 2003; Yamakawa et al., 2003). In addition, renin–angiotensin system activity has been associated with hyperactivity of the hypothalamic–pituitary–adrenal axis and may have direct neurotoxic effects that may contribute to depression (Vian et al., 2017). However, this study suggests that ACEI or ARBs that modulate systemic renin–angiotensin system activity do not have a protective effect on the development of depression in older individuals. The consistency of results across the primary and secondary endpoint and across several sensitivity analyses supports the robustness of the results. Further study is needed whether drugs that more specifically modulate renin–angiotensin system activity in the brain (e.g. brain aminopeptidase A inhibitors (Nakagawa et al., 2020)) may have neuroprotective effects.

ACEI and ARB initiators had a higher risk of depression compared to CCBs initiators. This may suggest a beneficial effect of CCBs on depression. In accordance, one small clinical trial (*n* = 101) showed that older individuals treated with fluoxetine combined with the CCB nimodipine reduced depressive symptoms more than treatment with fluoxetine alone (Taragano et al., 2005). Further study is needed to elucidate the effects of CCBs on depressive symptoms.

Strengths of this study include the use of an active comparator, new user design, and rigorous matching to reduce the risk of bias due to residual confounding. Our study also has several weaknesses. First, in the primary analysis, individuals were required to have previously used ⩾1 class of an antihypertensive drug other than an ACEI, ARB of thiazide(-like) diuretic before or at the index date. This selection of a specific group of individuals with hypertension may limit the generalizability of the study results. However, results were similar when we compared individuals irrespective of previous use of antihypertensive drugs. Second, we had no information on patient adherence to their prescribed drugs, which may lead to misclassifications of exposure. Such misclassification is likely nondifferential and may have led to underestimation of treatment effects. Third, reverse causality may have played a role, that is, there may be a time difference between the onset of depressive disorder and actual diagnosis. During this time period, there may be a higher chance of a new ACEI or ARB prescription. However, such bias will also have affected our control group of individuals using thiazide(-like) diuretics. Consistently, when we excluded individuals with a depression diagnosis within 30 days after the index date, the results were similar. Fourth, although we had a large sample size, the number of events was low in some stratified analyses and median duration of follow-up was relatively short. Fifth, this study included mainly older individuals with late-onset depression. The results may therefore not be generalizable to individuals with depression with onset earlier in life, and this requires further study. Sixth, we could not include in the analysis some potentially important covariates related to marital status or socioeconomic status, because data on these covariates are missing in a relatively large number of individuals in CPRD, as described previously (Jain et al., 2017). For example, marital status was missing in 81.8% of our matched cohort. Although considering one antihypertensive drug versus another is probably most strongly determined by the comorbidities or demographic characteristics that were taken into account in our analysis, and less profoundly by marital status, or socioeconomic status and other factors that influence access to medical care more broadly (Iadecola and Gottesman, 2019), we cannot exclude the possibility of residual confounding. Seventh, the primary outcome was broadly defined with the aim to include all cases of depression. However, this definition included fatal and nonfatal self-harm, and self-harm may be related to psychiatric conditions other than depression. Nevertheless, results of the analysis with the secondary outcomes treated or any depression (i.e. excluding fatal and nonfatal self-harm from the outcome) were consistent with the results of the main analysis.

In conclusion, in this large observational population-based study, the use of an ACEI or ARB compared with the use of negative control (thiazide(-like) diuretic use) was not associated with a lower risk of depression among older individuals.

## Supplemental Material

sj-docx-1-jop-10.1177_02698811221082470 – Supplemental material for Angiotensin-converting enzyme inhibitors and angiotensin receptor blockers and risk of depression among older people with hypertension Click here for additional data file. Supplemental material, sj-docx-1-jop-10.1177_02698811221082470 for Angiotensin-converting enzyme inhibitors and angiotensin receptor blockers and risk of depression among older people with hypertension by Thomas T van Sloten, Patrick C Souverein, Coen DA Stehouwer and Johanna HM Driessen in Journal of Psychopharmacology
