# Supplementary material for: Angiotensin-converting enzyme inhibitors and angiotensin receptor blockers and risk of depression among older people with hypertension
Source: J Psychopharmacol. 2022 Apr 7;36(5):594–603. doi: 10.1177/02698811221082470 (PMC9112619; doi:10.1177/02698811221082470)
Supplement: sj-docx-1-jop-10.1177_02698811221082470 – Supplemental material for Angiotensin-converting enzyme inhibitors and angiotensin receptor blockers and risk of depression among older people with hypertension [file sj-docx-1-jop-10.1177_02698811221082470.docx]

**DATA SUPPLEMENT**

**Angiotensin-converting enzyme inhibitors and angiotensin receptor blockers and risk of depression among people with hypertension**

TT van Sloten,^1,2^ PC Souverein,^3^ CDA Stehouwer,^1,2^ JHM Driessen^2,3,4,5^

1. Department of Internal Medicine, Maastricht University Medical Centre +, Maastricht, the Netherlands
2. School for Cardiovascular Diseases (CARIM), Maastricht University, Maastricht, the Netherlands
3. Division of Pharmacoepidemiology and Clinical Pharmacology, Utrecht Institute of Pharmaceutical Sciences, Utrecht University, Utrecht
4. Department of Clinical Pharmacy and Toxicology, Maastricht University Medical Centre +, Maastricht, the Netherlands
5. School for Nutrition, and Translational Research in Metabolism (NUTRIM), Maastricht University

**Table S1. Angiotensin-converting enzyme inhibitors, angiotensin receptor blockers, thiazide(-like) diuretic and calcium channel blockers included in the present study**

| **Angiotensin-converting enzyme inhibitors** | **Angiotensin receptor blockers** | **Thiazide(-like) diuretics** | **Calcium channel blockers** |
| --- | --- | --- | --- |
| Benazepril  Captopril  Cilazapril  Enalapril  Fosinipril  Imidapril  Lisinopril  Moexipril  Perindopril  Quinapril  Ramipril  Trandolapril | Azilsartan  Candersartan  Eprosartan  Irbesartan  Losartan  Olmesartan  Telmisartan  Valsartan | Bendroflumethiazide  Chlorothiazide  Chlorthalidone  Cyclopenthiazide  Hydrochlorothiazide  Hydroflumethiazide  Indapamide  Mefruside  Methyclothiazide  Metolazone  Xipamide | Amlodipine  Barnidipine  Benidipine  Felodipine  Isradipine  Lacidipine  Lercanidipine  Manidipine  Nicardipine  Nilvadipine  Nimodipine  Nifedipine  Nisodipine  Nitrendipine |

**Table S2: Lipophilic and less lipophilic angiotensin-converting enzyme inhibitors and angiotensin receptor blockers**

| **Lipophilic angiotensin-converting enzyme inhibitors or angiotensin receptor blockers** | **Less lipophilic angiotensin-converting enzyme inhibitors or angiotensin receptor blockers** |
| --- | --- |
| Azilsartan  Benazepril  Candersartan  Cilazapril  Fosinopril  Imidapril  Irbesartan  Moexipril  Olmesartan  Perindopril  Quinapril  Ramipril  Telmisartan  Trandolapril | Captopril  Enalapril  Eprosartan  Lisinopril  Losartan  Valsartan |

**Table S3. Incidence rates and hazard ratios for incident depression* for angiotensin-converting enzyme inhibitor (ACEI) or angiotensin receptor blocker (ARB) initiators compared to thiazide(-like) diuretic initiators – results of sensitivity analysis**

| **Exposures** | **Incidence rate per 1000 person-years (95%CI)** | **Absolute rate difference per 1000 person-years (95%CI)** | **Hazard ratio (95%CI)** |
| --- | --- | --- | --- |
| **Intention to treat analysis** | | | |
| Controls (thiazide-(-like) initiators) | 7.0 (6.4; 7.6) | 0 [Reference] | 1 [Reference] |
| ACEI/ARB initiators | 6.5 (6.0: 7.1 | -0.5 (-1.3 – 0.4) | 0.93 (0.83; 1.05) |
| **Exclusion of individuals with an event in the first 30 days of follow-up** | | | |
| Controls (thiazide-(-like) initiators) | 6.6 (5.7: 7.6) | 0 [Reference] | 1 [Reference] |
| ACEI/ARB initiators | 6.2 (5.5; 7.1) | -0.4 (-1.6 – 0.8) | 0.96 (0.79; 1.16) |
| **Selection of ACEI/ARB initiators vs thiazide(-like) diuretic initiators irrespective of previous use of antihypertensive drugs** | | | |
| Controls (thiazide-(-like) initiators) | 6.1 (5.6 – 6.8) | 0 [Reference] | 1 [Reference] |
| ACEI/ARB initiators | 6.2 (5.7 – 6.7) | 0.1 (-0.7 – 0.9) | 1.02 (0.90; 1.16) |

* Composite end point of treated depression and nonfatal and fatal self-harm.

ACEI = angiotensin converting enzyme inhibitor; ARB = angiotensin II receptor antagonist, CI = confidence interval.

| A: Propensity score for ACEI/ARB before matching | B: Propensity score for ACEI/ARB after matching |
| --- | --- |
| 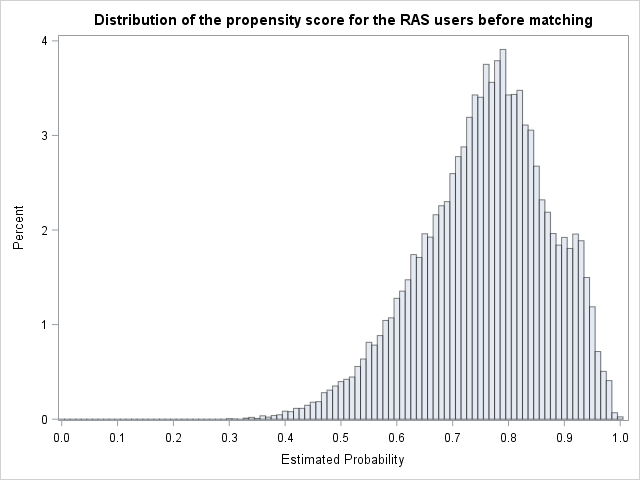 | 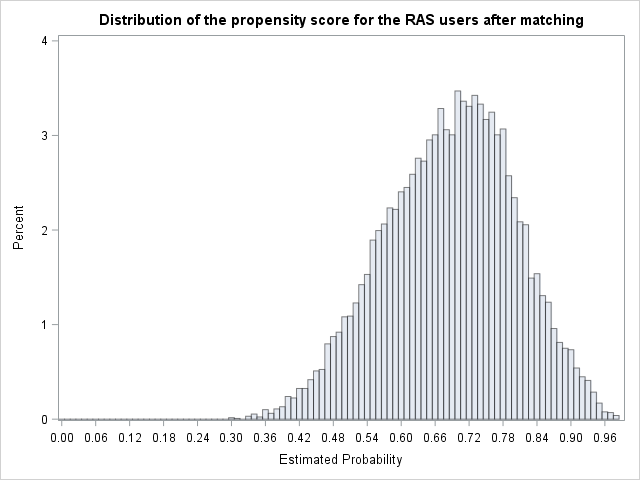 |
| C: Propensity score for thiazide(-like) diuretics before matching | D: Propensity score for thiazide(-like) diuretics before matching |
| 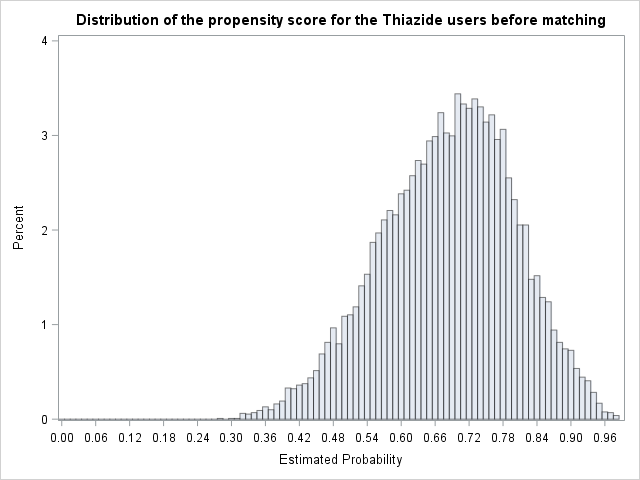 | 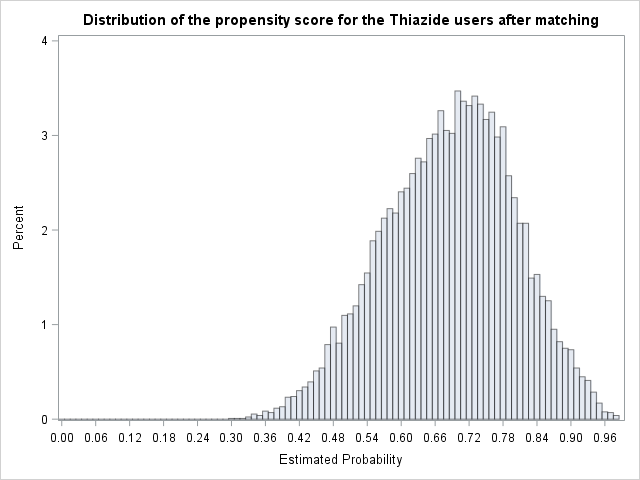 |

**Figure S1: Distribution plots of the propensity score before and after matching.**

ACEI = angiotensin converting enzyme inhibitor; ARB = angiotensin II receptor antagonist
